# Interferon-α-Conditioned Human Monocytes Combine a Th1-Orienting Attitude with the Induction of Autologous Th17 Responses: Role of IL-23 and IL-12

**DOI:** 10.1371/journal.pone.0017364

**Published:** 2011-02-28

**Authors:** Stefano M. Santini, Caterina Lapenta, Simona Donati, Francesca Spadaro, Filippo Belardelli, Maria Ferrantini

**Affiliations:** Department of Cell Biology and Neurosciences, Istituto Superiore di Sanità, Rome, Italy; French National Centre for Scientific Research - Université de Toulouse, France

## Abstract

IFN-α exerts multiple effects leading to immune protection against pathogens and cancer as well to autoimmune reactions by acting on monocytes and dendritic cells. We analyzed the versatility of human monocytes conditioned by IFN-α towards dendritic cell differentiation (IFN-DC) in shaping the autologous T-helper response. Priming of naïve CD4 T cells with autologous IFN-DC in the presence of either SEA or anti-CD3, resulted, in addition to a prominent expansion of CXCR3+ IFN-γ-producing CD4 Th1 cells, in the emergence of two distinct subsets of IL-17-producing CD4 T cells: i) a predominant Th17 population selectively producing IL-17 and expressing CCR6; ii) a minor Th1/Th17 population, producing both IL-17 and IFN-γ. After phagocytosis of apoptotic cells, IFN-DC induced Th17 cell expansion and IL-17 release. Notably, the use of neutralizing antibodies revealed that IL-23 was an essential cytokine in mediating Th17 cell development by IFN-DC. The demonstration of the IFN-DC-induced expansion of both Th1 and Th17 cell populations reveals the intrinsic plasticity of these DC in orienting the immune response and provides a mechanistic link between IFN-α and the onset of autoimmune phenomena, which have been correlated with both IL-17 production and exposure to IFN-α.

## Introduction

IFN-α has been recently recognized as an important factor in linking innate and adaptive immunity. In fact, through its rapid secretion by specialized cells, such as plasmacytoid DC (pDC), and concomitant capability to act on cells of innate and acquired immunity [1], [2], IFN-α promotes the induction of immune responses, including auto-reactive responses. In fact, IFN-α has been considered as the driving factor in several human autoimmune diseases such as Systemic Lupus Erythematosus (SLE) [3], myositis, Sjogren's syndrome and the initial phase of psoriasis [1].

IFN-α can contribute to the breaking of tolerance and to the induction/expansion of auto-reactive T and B cells through direct effects on these cells or by modulating immune functions through the induction of DC costimulatory molecules and soluble factors [1], [4]. Interestingly, the association between an increased production and or bioavailability of IFN-α and alterations in DC homeostasis has been indicated in various human inflammatory and autoimmune diseases. Of note, the Toll Like Receptor (TLR)-dependent production of IFN-α by pDC may play a central role in the pathogenesis of SLE [1], [3] and psoriasis [5] by inducing the differentiation of monocytes into highly activated myeloid DC.

The pathogenetic role of IFN-α in autoimmune disorders has been traditionally attributed to its ability to polarize T-helper cells toward the Th1 type of immune response and to induce the generation/activation of effector immune cells [6]. However, the discovery of IL-23 and IL-17 and the recognition of the new IL-23/Th17 axis in the pathogenesis of a variety of inflammatory and autoimmune diseases [7], [8] have led to reconsider the role played by cytokines other than IL-12, such as IFN-γ, TGF-β, IL-6, IL-1β, that are mainly produced by activated accessory cells and APC, particularly DC. In this regard, however, the possible role of IFN-α and DC in the induction of a Th17 CD4+ T cell response has not yet been investigated.

We as well as others [2, and references therein] have demonstrated that IFN-α induces the rapid differentiation of monocytes into highly active DC (IFN-DC), capable of promoting Th1 type immune responses through the expansion of CD4 and CD8 T cells producing large quantities of IFN-γ [9], [10]. Further studies suggested that cytokines belonging to the IL-12 cytokine family (IL-23 and IL-27), could play a role in the Th1-promoting activity of IFN-DC [10].

In this study, we have investigated the interaction between IFN-DC and autologous naïve CD4 T cells. The characterization of the cytokine milieu produced by IFN-DC revealed the release of soluble factors known to promote Th17 differentiation. Consistently, IFN-DC induced a clear-cut Th17 response and IL-17 production. Since Th17 lymphocytes have been implicated in the pathogenesis of autoimmune disorders, these findings are instrumental in understanding how IFN-α, by shaping DC functions including IL-23 release, can induce autoimmunity, thus leading to a more comprehensive vision of the role of this cytokine in the regulation of the immune response under both physiological and pathological conditions.

## Results

### IFN-α-conditioned DC and induction of a Th1 type response in the presence of SEA

In this study, two experimental systems were used in which human naïve CD4 T cells purified from peripheral blood were co-cultured with autologous IFN-DC or IL-4-DC and stimulated or not with either the superantigen SEA Vbeta specific [11], which activates a wide repertoire of T-cell receptors beta variable chains (TCRV beta) with subsequent massive proliferation of CD4 T cells, or anti-CD3-coated beads.

**Figure 1:**
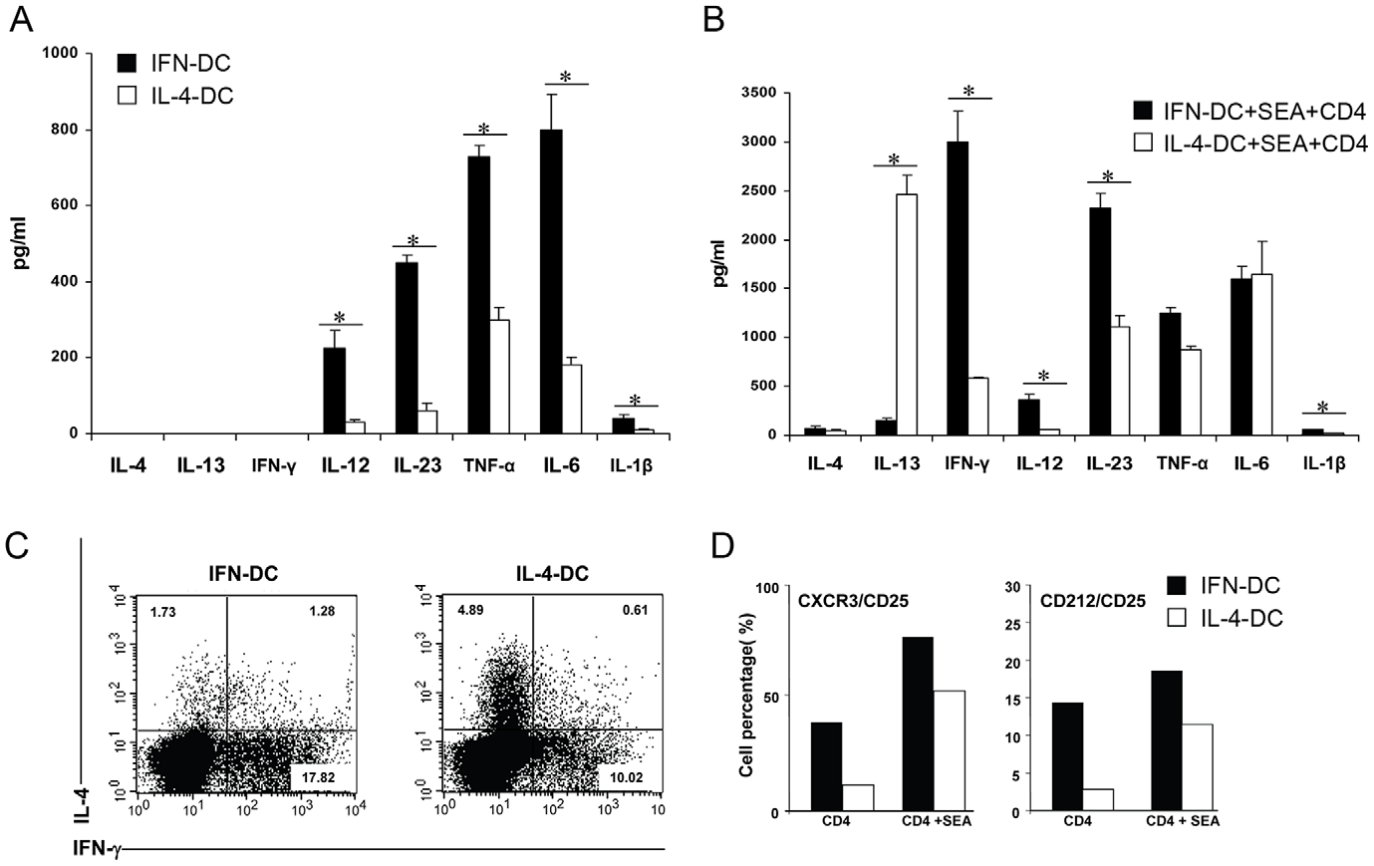
Cytokine production by IFN-DC and IL-4-DC and induction of a Th1 type response in naïve CD4 T cells stimulated with SEA in the presence of autologous DC. **A**) Cytokine production by IFN-DC and IL-4-DC. The amounts of the indicated cytokines were determined by ELISA in the DC culture supernatants, as described in Materials and Methods, after three days (IFN-DC) or five days of differentiation (IL-4-DC). The values represent the mean +/− SD of five independent experiments. Statistical analysis was performed by Mann-Whitney test. * *p*<0.05. **B**) Cytokine production in the supernatants of cultures of CD4 T cells stimulated with SEA in the presence of autologous IFN-DC or IL-4-DC. After six days of co-cultures, the cytokine production was analyzed by ELISA as described in Materials and Methods. The values represent the mean +/− SD of five independent experiments. Statistical analysis was performed by Mann-Whitney test. * *p*<0.05. **C**) Representative dot plot analysis of intracellular staining for IFN-γ and IL-4 in CD4 T cells primed with SEA in the presence of autologous DC. Naïve CD4 T cells were stimulated with SEA in the presence of autologous DC. At day 6 of co-culture, IFN-γ or IL-4 production at single cell level was analyzed, after activation with PMA/ionomycin in the presence of brefeldin-A as described in Materials and Methods, by intracellular cytokine staining. The analysis was performed on electronically gated CD4+ T cells. **D**) FACS analysis of the co-expression of CD212 and CD25 or CXCR3 and CD25 on CD4 T cells co-cultured with autologous DC for six days in the absence or presence of SEA. Each bar represents the percentage of double-positive CD4 T cells. Panels C and D show the results of one representative experiment out of three.

We preliminary evaluated the ensemble of cytokines accumulating in the supernatant from DC cultures. The analysis of IFN-DC cytokine milieu showed the presence, in addition to IL-12 and IL-23 as previously reported [10], of high levels of TNF-α and IL-6, together with lower levels of IL-1β (Fig. 1A). Significantly lower or virtually undetectable levels of these cytokines were secreted by IL-4-DC (Fig. 1A).

We then determined the profile and amount of cytokines released in the supernatant of co-cultures stimulated or not with SEA. As shown in Fig. 1B, in the absence of SEA the naïve CD4 T cells secreted significantly higher levels of IFN-γ when co-cultured with autologous IFN-DC as compared toIL-4-DC. Stimulation with SEA resulted in a substantial increase in the production of IFN-γ in the co-cultures of naïve CD4 T cells with either IFN-DC or IL-4-DC. However, stimulation in the presence of IFN-DC induced the release of much greater amounts of this cytokine. Considerable amounts of IL-13 were released by the naïve CD4 T cells co-cultured with IL-4-DC, whereas very low levels of this cytokine were secreted by the naïve CD4 T cells co-cultured with IFN-DC in the presence of the superantigen. Notably, significantly higher levels of IL-23 were secreted by IFN-DC as compared to IL-4-DC upon co-cultivation with autologous naïve CD4 T cells Intracellular IFN-γ and IL-4 staining performed on the expanded CD4 T cell population after restimulation with PMA/ionomycin, confirmed the explicit attitude of IFN-DC to strongly favor the development of IFN-γ-producing T cells (Fig. 1C). Likewise, the analysis of the co-expression of the surface markers CXCR3 or CD212 (IL-12Rβ1chain) with the activation marker CD25 confirmed that IFN-DC efficiently expanded *bona fide* Th1 cells (Fig. 1D).

**Figure 2:**
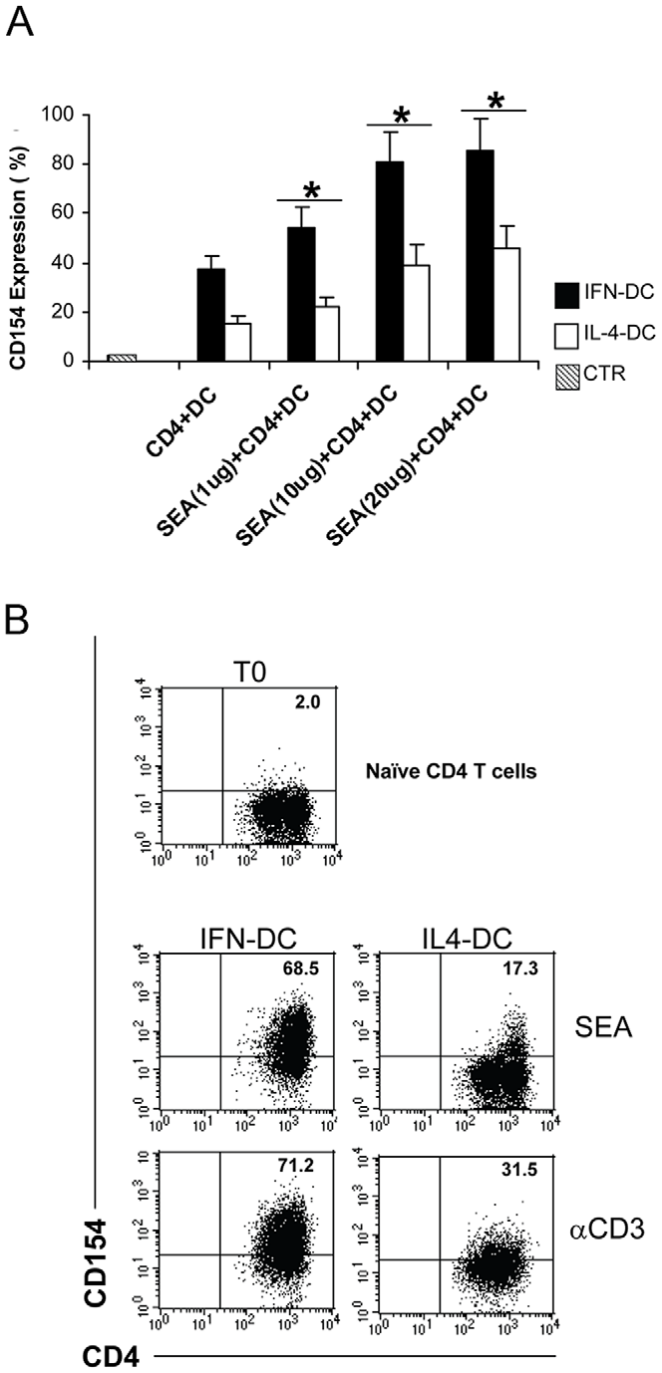
IFN-DC are superior with respect to IL-4-DC in inducing the expression of CD154 (CD40L). **A**) CD154 expression by naïve CD4 T cells following isolation (CTR) and after activation with different concentrations of SEA (1, 10, or 20 µg/ml) in the presence of autologous DC (1∶4 ratio) for 6 days. Each bar represents the mean percentage +/− SD of CD4 T cells expressing CD154, as derived from three independent experiments. Statistical analysis was performed by Mann-Whitney test. * *p*<0.05. **B**) Representative dot plot analysis of CD154 expression by CD4 T cells activated with either SEA or anti-CD3 in the presence of autologous DC for 6 days. The two analyses were performed on electronically gated CD4+ T cells.

Of note, IFN-DC were superior with respect to IL-4-DC in inducing the expression of CD154 (CD40L), a marker of activated CD4 T cells. At both intermediate and high SEA concentrations, a higher percentage of CD154+ T cells was detected when the naïve CD4 T cells were co-cultured with autologous IFN-DC as compared to IL-4-DC (Fig. 2A), and CD154 membrane expression was far more intense in the co-cultures with IFN-DC as compared to those with IL-4-DC, in the presence of either SEA or anti-CD3 (Fig. 2B). Replacement of SEA with anti-CD3 coated beads for naïve CD4 T cell activation resulted in a further increase in CD154 expression exclusively on CD4 T cells co-cultured with IL-4-DC, suggesting that IFN-DC provide *per se* an optimal co-stimulation for efficient priming of CD4 T cells and maximal CD154 expression (Fig. 2B).

### IFN-α-conditioned DC produce a Th17 phenotype-promoting cytokine environment

**Figure 3:**
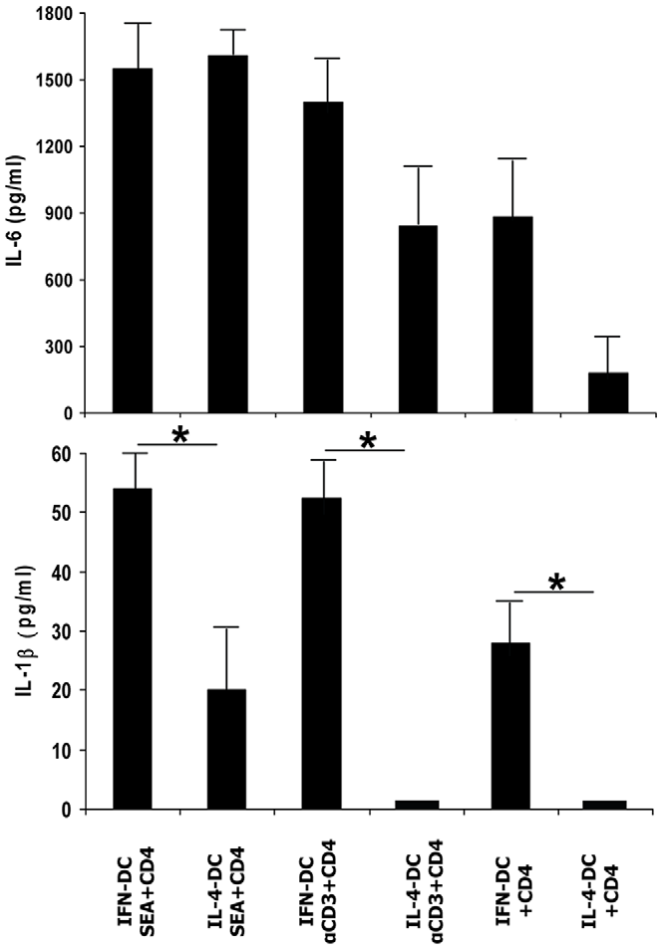
Release of IL-6 and IL-1β after co-culture of naïve CD4 T cells with autologous DC. The amount of IL-6 and IL-1β released in the co-cultures of naïve CD4 T cells with autologous DC, with or without activation with SEA or anti-CD3 coated beads, was determined by ELISA after 6 days of co-culture. The values represent the mean +/− SD of four independent experiments. Statistical analysis was performed by Mann-Whitney test. * *p*<0.05.

Notably, the profile of IFN-DC cytokine production was strongly suggestive of a Th17 phenotype-promoting milieu (Fig. 1A), as IL-1β, IL-6, and TNF-α, together with IL-23, have been found to induce and sustain Th17 development [12], [13]. In the light of the particular efficiency of IFN-DC in inducing high levels of CD154 in CD4 T cells (Fig. 2A and B) that are known to reciprocally activate DC through CD40-CD154 interaction, we then analyzed the cytokine production during the co-culture of naïve CD4 T cells with autologous DC in the absence or in the presence of either SEA or anti-CD3 coated beads, especially focusing on IL-6 and IL-1β, which are known to play a key role in Th17 development [13]. As shown in Fig. 3, considerable levels of IL-6 (upper panel) and IL-1β (lower panel) were found in the co-cultures of IFN-DC with naïve CD4 T cells in the absence of any stimulus, and a similar increase was induced upon stimulation with SEA or anti-CD3. In contrast, very low levels of IL-6 were secreted in the co-cultures of IL-4-DC with naïve CD4 T cells in the absence of stimuli, where IL-1β was virtually undetectable. An increase of IL-6 secretion in the co-cultures of naïve CD4 T cells with IL-4-DC occurred substantially in the presence of SEA and, at a lower extent, of anti-CD3, whereas IL-1β secretion was revealed only upon stimulation with SEA. Large amounts of TNF-α, ranging from 1,159 pg +/− 306 to 1,592 +/− 254 (mean +/− SD values), were invariably present in all culture conditions and with both types of DC, while TGF-β was in any case undetectable (data not shown).

### IFN-DC-driven induction of Th17 CD4 T cells

**Figure 4:**
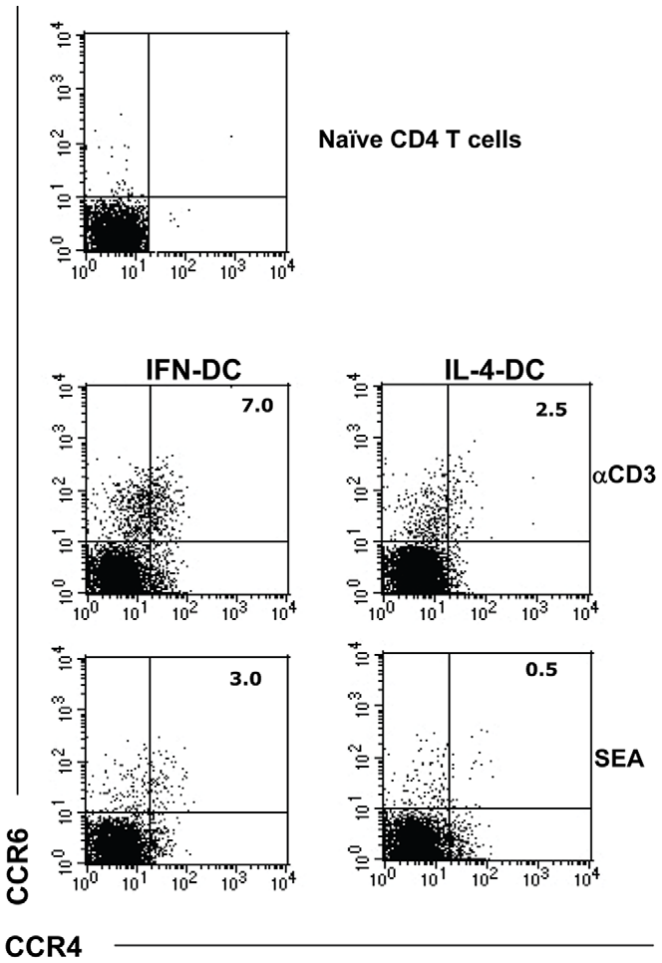
Estimation of the emergence of Th17 cells by analysis of surrogate cell marker expression. Dot plot analysis of the expression of CCR6 and CCR4 on CD4 T cells after a 6-day stimulation with SEA or anti-CD3 coated beads in the presence of autologous DC. The results of one representative experiment out of five independently performed are shown. In all cases, the analysis was performed on electronically gated CD4+ T cells.

In the light of the cytokine milieu produced by IFN-DC, we evaluated whether these APC could favor the emergence of Th17 CD4 lymphocytes. In this set of experiments, anti-CD3-coated beads were used to stimulate the naïve CD4 T cells in the co-cultures with autologous DC, to obtain a higher percentage of responding CD4 T cells and amplify the phenomenon. Remarkably, the FACS analysis demonstrated the concomitant expression of CCR4 and CCR6 (Fig. 4), that is assumed to identify the Th17 CD4 subset [12], [13], on a fraction of CD4 T cells primed in the presence of autologous IFN-DC.

**Figure 5:**
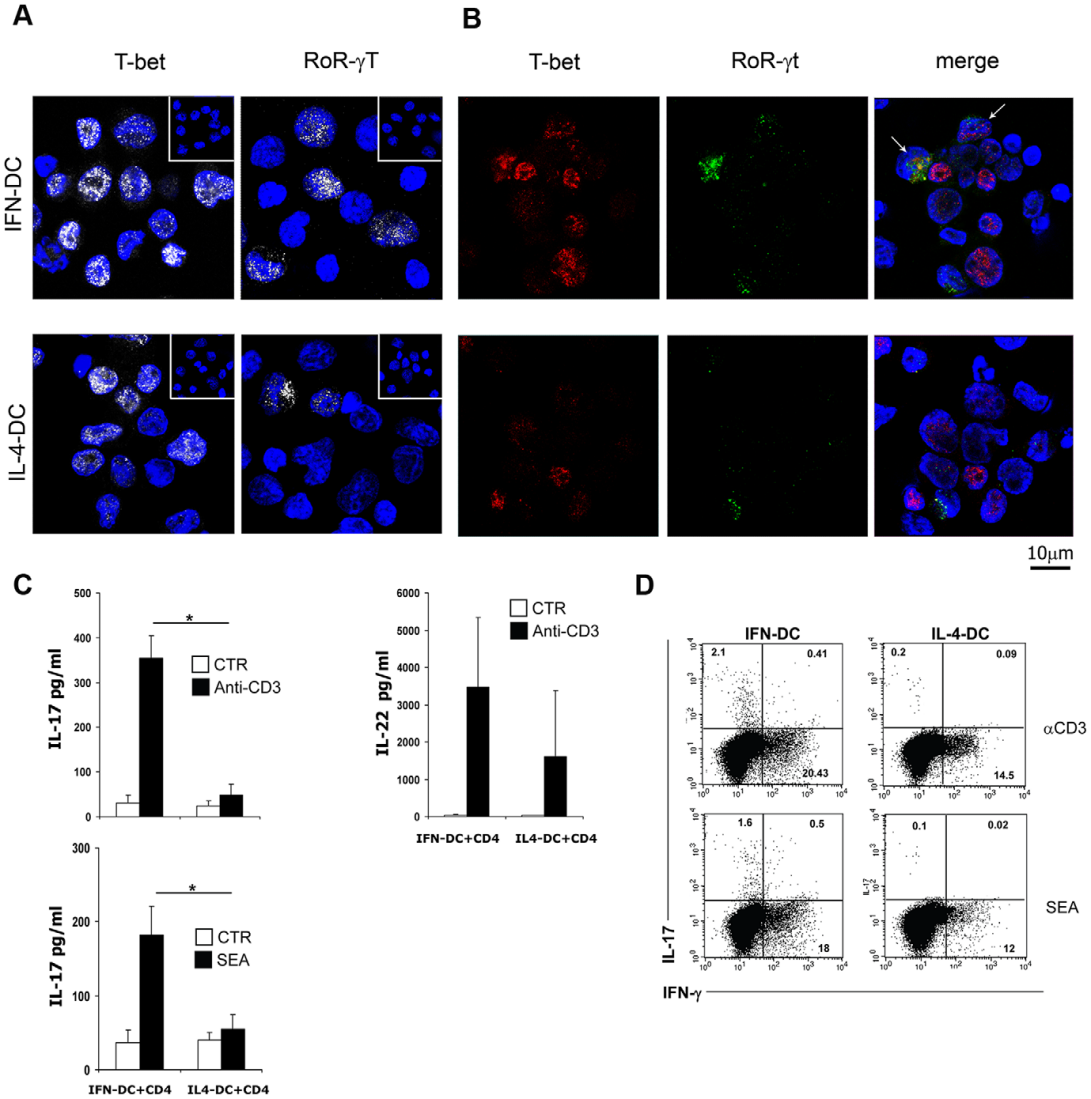
Expression of T-bet and RoR-γt transcription factors. IL-17 and IL-22 production by CD4 T cells stimulated in the presence of IFN-DC. **A**) CLSM analysis (central optical sections) was performed on CD4 T cells activated by anti-CD3-coated beads in the presence of autologous DC for six days to evaluate the expression of the transcription factors T-bet and RoR-γt (pseudo-color grey). Cell nuclei were stained with DAPI (blue). Insets represent background labelling of isotype control antibodies. One representative experiment out of three independently performed is reported. **B**) Co-expression of the transcription factors T-bet (red) and RoR-γt (green) in CD4 T cells activated with anti-CD3-coated beads in the presence of autologous DC for six days, as observed in a representative experiment out of three. Cell nuclei were stained with DAPI (blue). One representative experiment out of three independently performed is reported. **C**) Production of IL-17 and IL-22 in the culture supernatants of CD4 T cells stimulated in the presence of autologous DC. Naïve CD4 T cells were stimulated with SEA or anti-CD3 in the presence of autologous DC for six days. The values represent the mean +/− SD of four independent experiments. Statistical analysis was performed by Mann-Whitney test. * *p*<0.05. **D**) Production of IL-17 by a fraction of CD4 T cells, as demonstrated by intracellular cytokine staining. The dot plot analysis, representative of one out of five experiments, shows the intracellular IFN-γ and L-17 expression by CD4 T cells primed with SEA or anti-CD3 in the presence of autologous DC for six days. Upon activation with PMA/ionomycin in the presence of brefeldin-A for 5 hours, as described in Materials and Methods, cells were stained for the CD4 antigen, permeabilized and stained for intracellular IFN-γ and IL-17. Analysis was performed on electronically gated CD4+ T cells.

We have then assessed by CLSM analysis the expression of the transcriptional factors T-bet and RoR-γt, specific for the Th1 and Th17 subsets, respectively. The majority of CD4 T cells activated in the presence of autologous DC selectively expressed T-bet, while RoR-γt was detected in only a minority of the cells (Fig. 5A, B). Consistently with the results indicating the IFN-DC-induced expansion of Th17 cells, the percentage of CD4 T cells expressing RoR-γt was higher in the cultures stimulated with anti-CD3-coated beads in the presence of autologous IFN-DC (mean 9.7±SD 1.8%) than in the co-cultures with IL-4-DC (mean 2.5±SD 2.4%) (Fig. 5A, B). The co-expression of T-bet and RoR-γt was detected in about 1% of CD4 T cells expanded by anti-CD3-coated beads and IFN-DC, whereas it was virtually absent in the CD4 T cell-IL-4-DC co-cultures (Fig. 5B).

The production of IL-17 was evaluated after six days of CD4 T cell-DC co-culture in the presence of SEA or anti-CD3-coated beads by ELISA (Fig. 5C, top and middle panel) and by intracellular IL-17 staining to detect and quantify IL-17-producing cells by FACS analysis (Fig. 5D). The ELISA results clearly demonstrated the release of significantly higher amounts of IL-17 upon SEA or anti-CD3 stimulation of CD4 T cells in the presence of autologous IFN-DC, as compared to the co-culture with IL-4-DC. Considerable levels of IL-22, cytokine also associated to the Th17 phenotype, were secreted after stimulation of naive CD4 T cells with anti-CD3-coated beads in the presence of either type of autologous DC (Fig. 5C, bottom panel).

Intracellular staining for the detection of IFN-γ and IL-17 after restimulation of the expanded CD4 T cells with PMA/ionomycin, confirmed not only the efficiency of IFN-DC in promoting the expansion of IFN-γ-producing Th1 cells, but also their capability to induce the development of IL-17-producing T cells (Fig. 5D). A small but clearly detectable fraction of cells producing both IL-17 and IFN-γ was identified in the cultures of CD4 T cells stimulated with SEA or anti-CD3-coated beads in the presence of IFN-DC (Fig. 5D). We could not detect any expansion of Th17 cells nor IL-17 production when naïve CD4 T cells were activated with anti-CD3 plus anti-CD28 in the presence of IL-2 and IL-23 (data not shown), ruling out the possibility of the outgrowth of contaminating memory Th17 cells.

### Role of IFN-DC-secreted cytokines in the induction of Th17 cell development

**Figure 6:**
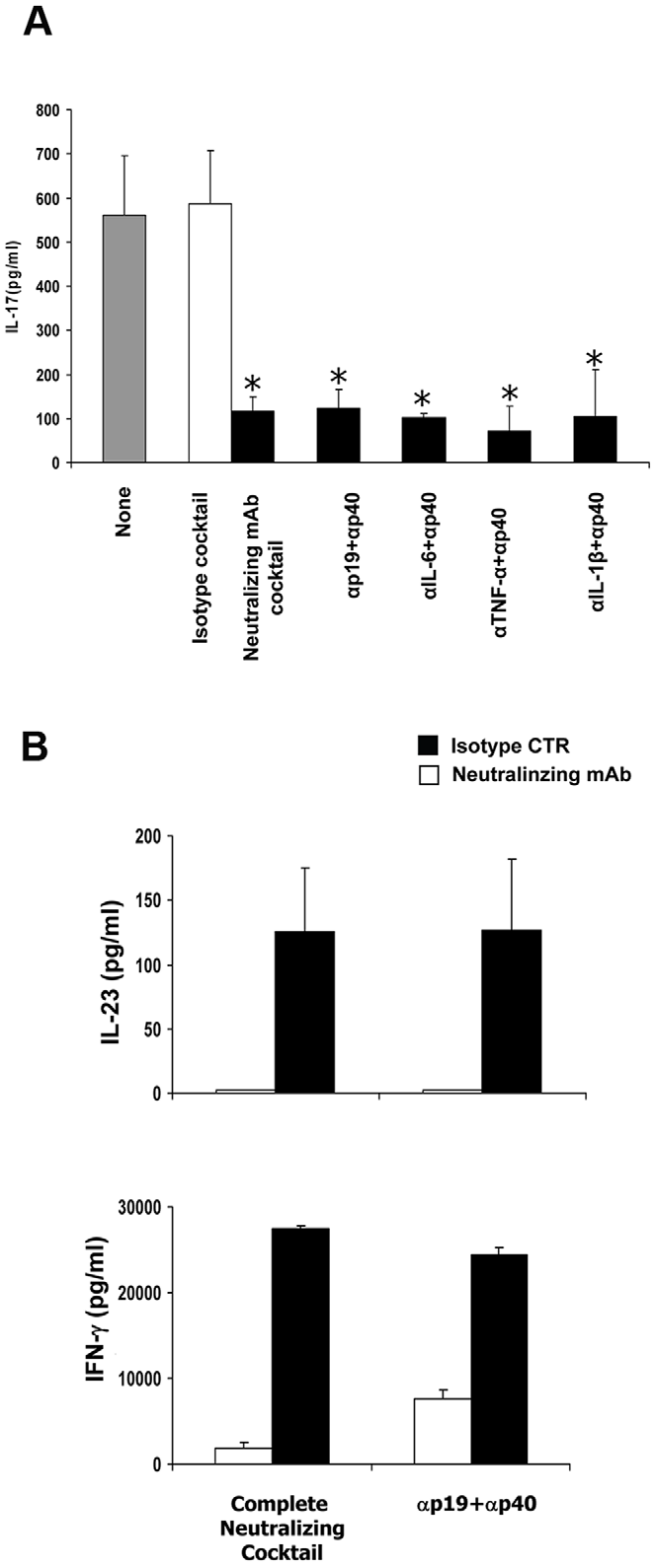
Inhibition of IL-17 production by selective cytokine neutralization. **A**) Inhibition of IL-17 production by selected couples or cocktail of cytokine blocking antibodies. Naïve CD4^+^ T cells were activated with anti-CD3 coated beads and autologous DC at a cell ratio of 1∶4 in the presence of the indicated couples of cytokine-neutralizing antibodies, or of the respective control isotypes. The presence of IL-17 was analyzed in the supernatants of cell cultures after 6 days by ELISA. The effect of the control isotypes couples on IL-17 production was not significantly different from that of the control isotypes cocktail. The values represent the mean +/− SD of three independent experiments. Statistical analysis was performed by Mann-Whitney test. * *p*<0.05. **B**) Inhibition of IFN-γ and IL-23 production by selective IL-23/IL-12 neutralization as compared to the inhibition obtained with the complete neutralizing cocktail, including antibodies to the p40 subunit of IL-12 and IL-23, p19 (IL-23), IL-1β, TNF-α and IL-6. Th values represent the mean +/− SD of three independent experiments. Statistical analysis was performed by Mann-Whitney test. * *p*<0.05.

We then asked which IFN-DC cytokines were required for IL-17 induction in naïve CD4 T cells. As expected, the antibody blockade of an ensemble of cytokines (i.e. IL-1β, IL-6, TNF-α, and IL-12/IL-23) markedly inhibited IL-17 production (Fig. 6A), whereas neutralization of single cytokines did not significantly prevent IL-17 production (data not shown). Interestingly, neutralizing antibodies against the IL-23p19 and the IL-12/IL-23p40 consistently and markedly inhibited IL-17 production in the co-cultures of naïve CD4 T cells with IFN-DC (Fig. 6A). The combination of anti-p19 and anti-p40 antibodies was used to obtain the highest possible neutralization of IL-23 as, in our experimental setting, the addition of the anti-p19 antibody alone reproducibly resulted in only a limited neutralization of IL-23 and a slight inhibition of IL-17 production (data not shown). In contrast, the addition of both anti-p19 and anti-p40 antibodies resulted not only in a virtually complete disappearance of IL-23 (Fig. 6B, upper graph), but also in a sharp reduction of IFN-γ production in the co-cultures of naïve CD4 T cells with autologous IFN-DC (Fig. 6B, lower graph). No significant increase in the inhibition of IL-17 production was observed when anti-IL-1β, anti-IL-6 or anti-TNF-α neutralizing antibodies were added together with the anti-p40 antibodies to the anti-CD3-activated naïve CD4 T cell-IFN-DC co-cultures (Fig. 6A). Overall, these results together with the observation that much higher levels of IL-23 as compared to IL-12 were reproducibly detected in those co-cultures (data not shown), strongly indicate IL-23 as an essential cytokine in mediating human Th17 cell development by IFN-DC.

### IFN-DC phagocytosis of apoptotic cells induces the production of Th17 inducing cytokines and IFN-γ

A recent study demonstrated that in mouse models the phagocytosis of apoptotic cells by DC in the presence of infection-induced signals promotes Th17 cell differentiation [14].

**Figure 7:**
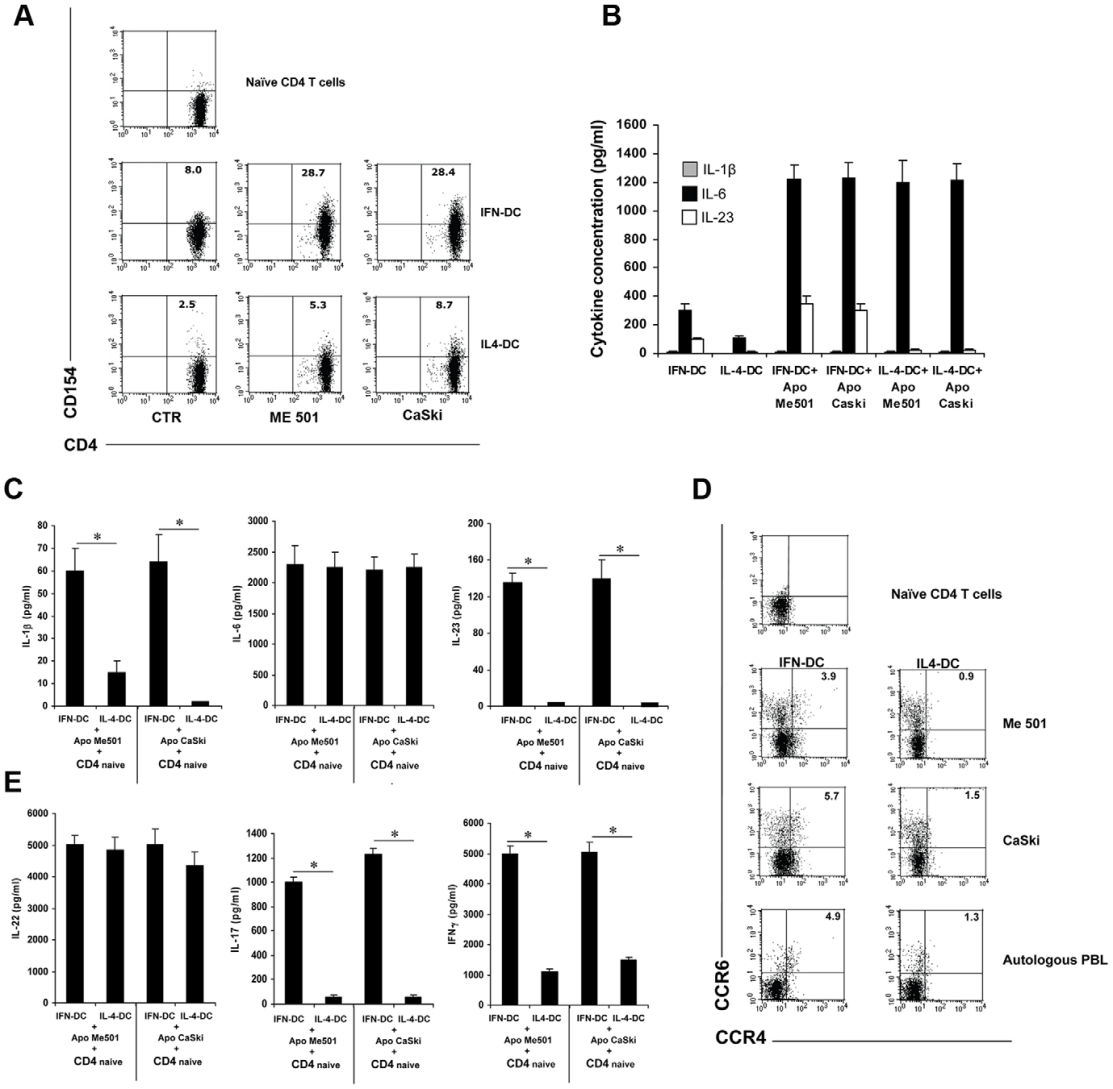
Induction of Th17 response by DC loaded with apoptotic cells. **A**) Representative dot plot analysis (one out of three) of CD154 induction in CD4 T cells after 6 days of culture with autologous IFN-DC or IL4-DC fed with apoptotic cells. The upper dot plots show CD154 expression in purified naïve CD4 T cells prior to use. CTR dot plots show the percentage of CD154 expression by CD4 T cells cultivated in the presence of autologous DC which had not phagocytosed apoptotic tumor cells. Analysis was performed on electronically gated CD4+ T cells. The percentage of early and late apoptotic tumor cells was evaluated by Annexin-V/propidium iodide staining. **B**) ELISA detection of IL-1β, IL-6 and IL-23 release by DC upon phagocytosis of apoptotic tumor cell lines. Analysis was performed after 18 hours from tumor cell uptake. The values represent the mean +/− SD of three independent experiments. **C**) ELISA detection of IL-1β, IL-6 and IL-23 in supernatants from co-culture of CD4 T cells with autologous DC which had phagocytosed apoptotic cells. After three days of co-culture, CD4 T cells were restimulated overnight with anti-CD3 coated beads. At day four the supernatant was collected for cytokine detection. All data are expressed as the mean +/− SD of three independent experiments. Statistical analysis was performed by Mann-Whitney test. * *p*<0.05. **D**) Dot plot analysis of CCR6/CCR4 expression by CD4 T cells after six days of stimulation in the presence of DC fed with apoptotic tumor cells or autologous PBL. The results of one representative experiment out of three are shown. In all case, the analysis was performed on electronically gated CD4+ T cells. **E**) Evaluation of IL-22, IL-17 and IFN-γ production at day 4, after overnight restimulation of CD4 T cells with anti-CD3 coated beads. All data are representative of one out of three independent experiments performed with cells derived from different donors. The values represent the mean +/− SD of three independent experiments. Statistical analysis was performed by Mann-Whitney test. * *p*<0.05.

To investigate whether apoptotic cell-loaded IFN-DC could promote the expansion of IL-17-secreting cells, we cultivated naïve CD4 T cells with autologous DC which had phagocytosed apoptotic melanoma (Me501) or cervical cancer (CaSki) cells. Interestingly, a notable increase in the percentage of CD154+ cells was induced by the co-cultivation of naïve CD4 T cells with apoptotic cell-loaded IFN-DC (Fig. 7A). As shown in Fig. 7B, the uptake of apoptotic tumor cells induced a massive production of IL-6 in both IFN-DC and IL-4-DC, while the production of IL-23 was selectively induced in IFN-DC. On the contrary, only irrelevant levels of IL-1β could be detected in all the conditions tested. Upon culture of naïve CD4 T cells with autologous DC loaded with apoptotic cells, high levels of IL-6 were found in the supernatants of all the different DC-CD4 T cells co-cultures, whereas IL-1β and IL-23 were produced mainly in the co-cultures of CD4 T cells with apoptotic cell-loaded IFN-DC (Fig. 7C). High levels of TNF-α were present in all culture conditions, while TGF-β was in all cases undetectable (data not shown).

Notably, the expansion of CCR6/CCR4 double positive cells clearly induced by stimulation with apoptotic cell-loaded IFN-DC (Fig. 7D), correlated with the levels of IL-17 detected in the corresponding culture supernatants (Fig. 7E), indicating the capability of IFN-DC which had phagocytosed apoptotic tumor cells to drive Th17 differentiation. Significantly higher levels of IFN-γ were induced following stimulation of naive CD4 T cells with apoptotic cell-loaded IFN-DC as compared to IL-4-DC counterparts. In contrast, high levels of IL-22 production were detected in all conditions.

An expansion of CCR6/CCR4 double positive cells was found to occur also upon stimulation of naive CD4 T cells with IFN-DC fed with apoptotic autologous PBLs (Fig. 7D), arguing against the possibility that the Th17-inducing capability of apoptotic cell-loaded IFN-DC was dependent on tumor cell-specific factors.

## Discussion

Here we have shown that IFN-α-conditioned DC can act as effective APC in driving the development of Th17 cells from autologous naïve CD4 T cells. In fact, the priming of naïve CD4 T cells with autologous IFN-DC in the presence of either SEA or anti-CD3, resulted, in addition to a prominent expansion of CXCR3+ IFN-γ-producing CD4 Th1 cells, in the emergence of two distinct subsets of IL-17-producing CD4 T cells: i) a predominant Th17 population selectively producing IL-17 and expressing CCR6; ii) a minor Th1/Th17 population, producing both IL-17 and IFN-γ (Fig. 5D).

The exact requirements of individual cytokines or their combination for human Th17 differentiation are still controversial [7]. Recent reports have indicated an important role of IL-1β, IL-6 and IL-23 in human Th17 differentiation [7], [8], [12]–[17]. Notwithstanding, human IL-17A-producing cells have been recently demonstrated to originate from CD161+ CD4+ T-cell precursors upon activation in the presence of IL-1β plus IL-23. The essential role of IL-23 either in inducing IL-17 production by human T cells [18] or in promoting the maintenance rather than the priming of the IL-17-secreting cell population has been also demonstrated [19]. However, the *in vivo* cytokine requirements for human Th17 differentiation are still matter of investigation, particularly under the physiological setting of naïve CD4 T cells encountering antigen-presenting DC in the lymph nodes. Recent reports demonstrated that human DC differentiated *in vitro* from monocytes and activated through TLR [20] or inflammatory cytokines [21] were more efficient APC as compared to monocytes in inducing the expansion of IL-17-secreting cells upon stimulation of either allogeneic [20] or autologous T cells [21].

Here, we demonstrate, for the first time, that IFN-α-conditioned monocyte-derived DC act as highly efficient APC in inducing autologous IL-17-secreting Th17 cells, and that this effect is mediated by IL-23 and IL-12, as clearly indicated by the results of the neutralization experiments (Fig. 6). Our observation that much higher levels of IL-23 as compared to IL-12 were reproducibly detected in the cultures of naïve CD4 T cells stimulated with anti-CD3-coated beads in the presence of IFN-DC (data not shown), strongly support the predominant role of IL-23 in mediating human Th17 cell development by IFN-DC. Although our results argue against an essential role of IL-1β in the IFN-DC-induced expansion of IL-17-secreting cells, we cannot rule out that the low basal levels of IL-1β secreted by IFN-DC are indeed playing an important role in the priming of naïve CD4 T cells towards the IL-17-secreting phenotype, as also suggested by a recent report [20]. Notably, the IFN-DC capability to promote a Th17 response may also reside on their particular efficiency in inducing the expression of the CD40 ligand CD154 (Fig. 2 and 7A), as CD40-CD40L cross-talk has been recently reported to play a role in the establishment of the Th17 response [22].

**Figure 8:**
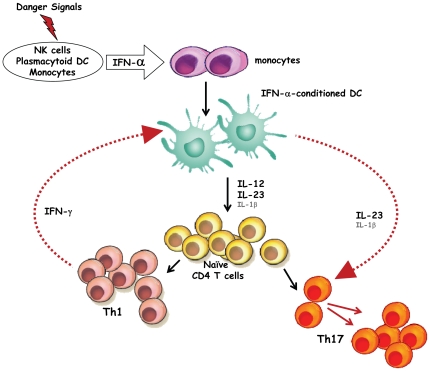
Model on the induction of Th17 cell development by IFN-α-conditioned DC. Distinct cell types, such as monocytes, NK cells and pDC can produce biologically relevant quantities of type I IFNs under different stimulation conditions, such as danger signals represented by a wide array of inflammatory cytokines and molecules derived from viruses and bacteria. In particular, pDC are responsible for producing very high levels of IFN-α in response to viral infections. Endogenous IFN-α production affects several immune functions and act by promoting DC differentiation from circulating monocytes and DC activation. These IFN-α-conditioned DC, which may represent the *in vivo* counterpart of IFN-DC, produce a wide array of inflammatory cytokines including IL-12, IL-1β and IL-23 which act on naïve CD4 T cells sustaining their differentiation into Th1 or Th17 cells. The IFN-γ produced at high levels by the Th1 cells enhances the APC production of IL-1β and IL-23, which, in turn, act on both Th1 and Th17 memory cells, promoting their expansion. In this scenario, IL-12 would act in the initial phase by priming the Th1 response, whereas IL-23 would contribute to the maintenance of both Th1 and Th17 memory cells.

Interestingly, at variance with previous reports [23], [24], the high levels of IFN-γ secreted upon stimulation of naïve CD4 T cells with IFN-DC did not appear to abrogate the capability of IFN-DC to induce the differentiation of Th17 cells from naïve CD4 T cell precursors. Two opposite effects of IFN-γ on APC have been recently identified: the inhibition of the capacity to polarize the immune response towards the Th1 profile and the induction of IL-1β and IL-23 production, resulting in the expansion of memory Th17 cells [25]. The novel pathway of APC-mediated T cell differentiation indicated by our study is fully consistent with the dynamic model proposed by Kryczek and coworkers [25]. In particular, the ensemble of our data unravel a new scenario in which IFN-α, cytokines produced early in response to viral infections or other danger signals, can rapidly induce a polyfunctional DC type, i.e. the *in vivo* counterpart of IFN-DC, capable of secreting IL-12, IL-1β and IL-23 and concomitantly inducing the emergence of Th1, Th17 and Th1/Th17 CD4 T cells. The IFN-γ produced by the differentiated Th1 cells may trigger the further production of IL-23 by IFN-DC, promoting the expansion of memory Th17 cells. This scenario (Fig. 8) is consistent with the observation that the levels of IL-23 strongly increase when IFN-DC are co-cultured with activated CD4 T cells (Fig. 1B, 3B, and 7B), as a consequence of IFN-γ production induced by IFN-DC-secreted IL-12 and IL-23. Of note, in our study the neutralization of IL-12 and IL-23 resulted in a sharp reduction of IFN-γ secretion in the co-culture of naïve CD4 T cells with autologous IFN-DC (Fig. 6). Taking into consideration the rapid exhaustion of IL-12 that we observed in the co-cultures of CD4 T cells with autologous IFN-DC (data not shown), it can be envisaged that, in the above proposed scenario, the IL-12 would act in the initial phase by priming the Th1 response, whereas the IL-23 would contribute to the maintenance of both the Th1 and the Th17 memory subsets, in agreement with previous reports [26], [27].

The capability of IFN-DC to expand both a Th1 and a Th17 population is consistent with the new concept that both these subsets play a role in the pathogenesis of inflammatory and autoimmune diseases. The IFN-DC-mediated induction of CD4 T cells co-expressing IFN-γ and IL-17, the Th1/Th17 cells, is also interesting in the light of the evidence that in patients with certain autoimmune diseases (e.g. Crohn disease, uveitis), a considerable fraction of T cells secreting IL-17 in the tissue was described to express both IFN-γ and IL-17 [28], [29].

Both IL-17 and IFN-α have been implicated in the pathogenesis of some autoimmune and inflammatory diseases. SLE represents a prototypic autoimmune disease characterized by a break of tolerance to self molecules, for which the possible pathogenetic role of IFN-α has been strongly emphasized [3]. Moreover, the seminal work by Banchereau's group demonstrated that the IFN-α contained in the sera from SLE patients induces the differentiation of circulating monocytes into APC strongly resembling IFN-DC [30].

Rheumatoid arthritis (RA) [31], Crohn's disease [32], [33], and psoriasis [34] are other autoimmune inflammatory conditions in which an IFN-α/IL-23/IL-17 axis may be involved. Interestingly, activated DC infiltration has been shown in Crohn's samples [32] with IL-23 mediating intestinal inflammation [33].

Our results clearly demonstrate that in an autologous human *in vitro* setting IFN-α acts on monocytes inducing their differentiation into APC capable of promoting the induction and expansion of both Th1 and IL-17-secreting, i.e. Th17 and Th1/Th17, subsets from naïve CD4 T cells.

IFN-α represents the cytokine exhibiting the longest record of use in clinical oncology and many data support the concept that the antitumor response observed in IFN-treated cancer patients is mediated by an activation of immune cells, including T lymphocytes [6] and DC [35]. Intriguingly, in melanoma patients, a striking correlation between the clinical response and the development of signs of autoimmune reactions has been demonstrated [36]. Notably, autoimmunity and effective tumor immunity often accompany one another, strongly suggesting that autoimmunity is a correlate of therapeutic benefit [37]. In the light of all this and of the recent evidence that Th17 cells play a role in immune-mediated control of tumors [38], [39], [40], our results may provide a mechanistic interpretation for the observed coupling of protective antitumor immunity with autoimmunity during IFN-α therapy. In fact, in previous studies we demonstrated that IFN-DC can effectively mediate the cross-priming of IFN-γ-secreting CD8 T cells against exogenous antigens [10], including apoptotic cell-derived antigens [41]. In the present study, we show that IFN-DC can also prime a Th17 response upon uptake of apoptotic cell-derived materials from tumor cells or autologous PBLs. Thus, we can assume that IFN-DC developing *in vivo* under pathological conditions or during IFN-α therapy can take-up apoptotic bodies from tumor cells and self cells as well, and can prime both Th1 and Th17 responses, thus leading to the expansion and activation of effectors T cells reactive against tumor-associated and self antigens.

The main research challenge in cancer immunotherapy is the development of effective and selective antitumor response, limiting immune attack to normal tissues. The understanding of how IFN-DC can induce a Th17 cell response can open new perspectives for the treatment of some autoimmune diseases and for the development of more effective immunotherapy strategies in cancer patients.

## Materials and Methods

### Ethic Statement

Peripheral blood mononuclear cells utilized in this study derived from buffy coats obtained from healthy blood donors, as anonymously provided by the Immunohematology and Transfusional Center of Policlinico Umberto I, University “La Sapienza”, Rome. Written informed consent for the use of buffy coats for research purposes was obtained from blood donors by the Transfusional Center and both the informed consent form and procedure was approved by the Ethics Committee of Policlinico Umberto I. Data related to human samples were all analyzed anonymously.

### Cell preparation

Peripheral blood mononuclear cells were purified by Ficoll density gradient centrifugation (Biochrom AG). Monocytes were isolated by immunomagnetic positive selection (MACS Cell Isolation Kits; Miltenyi Biotec) and plated at the concentration of 2×10^6^ cells/ml in RPMI medium (GIBCO BRL), supplemented with 10% FBS, 500 U/ml GM-CSF (Peprotech) and either 250 U/ml IL-4 (R&D Systems) for 5 days or 10,000 U/ml IFN-α2b (Intron A; Shering-Plough) for 3 days. Negatively selected PBLs were used to isolate naïve CD4+ T cells by negative selection using the Naïve CD4 T cell isolation kit II (Miltenyi Biotec). The isolated naïve CD4+ T cells proved to be at least 95% pure.

### Cytokine assays

Cytokine quantification was performed by commercial ELISA kits for: IL-1β, IL-4, IL-6, IFN-γ, TNF-α IL-12 (Endogen), IL-23 (Bender MedSystem), IL-13, IL-17, IL-22, TGF-β1 (R&D Systems) according to manufacturer instruction.

### CD4+ T cells activation

DC, obtained as described above, were washed and cultured with autologous naïve CD4+ T cells (1∶4 ratio) and 1 µg/ml SEA (Staphylococcal enterotoxin A, Sigma-Aldrich) for 6 days at 37°C, or anti-CD3 coated beads (T cell activation/Expansion Kit. Miltenyi Biotech), using the manufacturer's recommendations.

### FACS analysis of surface markers and intracellular cytokine expression

Cells were stained with a panel of fluorochrome-conjugated mAbs (BD Bioscience, R&D Systems) specific for T cells (anti-CD4, anti-CD25, anti-CXCR3, anti-CD212, anti-CCR6, anti-CCR4, anti-CD154).

Cytokine staining was performed after 6 days of DC/naïve T cells culture (1∶4 ratio). Cells were stimulated for 5 h with PMA (40 ng/ml) and ionomycin (500 ng/ml) (Sigma-Aldrich), in the presence of Golgi Plug (1 µg/ml) (BD Pharmingen) for the final 4 h of culture at 37°C. Cells were fixed and permealized with BD Cytofix/Cytoperm Plus (BD Biosciences Pharmingen) and incubated at 4°C for 30-min with FITC-labeled anti IFN-γ (BD Pharmingen), PE-labelled anti IL-4 (BD Pharmingen), or PE-labelled anti IL-17 (eBioscience), washed and analyzed on a FACScalibur cytometer with CellQuest software (Becton Dickinson).

### Confocal laser scanning microscopy (CLSM) analysis

CD4 T cells were seeded on cover glasses coated with poly-L-lysine (Sigma-Aldrich) Cells were fixed by paraformaldehyde (PFA) 3% (30 min, 4°C), permeabilized by Triton X-100 (0.5% 10 min at room temperature, Sigma-Aldrich) and then stained at 37°C with phycoerythrin (PE) anti mouse T-Bet Ab (eBioscience) and/or with rabbit polyclonal anti RoR-γt Abs (ABCAM), followed by Alexa Fluor 488 conjugated goat anti rabbit Abs (Molecular Probes). CLSM observations were performed with a Leica TCS SP2 AOBS apparatus. Image acquisition and processing were carried out using the Leica Confocal Software (Leica Lasertechnik). At least 1000 cells were analyzed in each field.

### Neutralization Assay

DC were cultured with autologous naïve CD4+ T cells at a ratio of 1∶4 and anti-CD3 activation beads (Milteny Biotech), in the presence of the following neutralizing antibodies: anti-IL-1β (10 µg/ml), anti-IL-6 (10 µg/ml), anti-TNF-α (10 µg/ml) (eBioscience), anti-IL-23p19 (10 µg/ml) (R&D Systems), anti-IL-12p40 (10 µg/ml) (Abcam), or the respective isotypic controls Abs. IL-17, IFN-γ and IL-23 production was analyzed after 6 days by ELISA (R&D Systems).

### Induction of Th17 response by DC loaded with apoptotic tumor cells

DC were loaded with apoptotic Me501 (melanoma) and CaSki (cervical cancer) tumor cells prior to co-culture. Apoptosis of Caski or Me501 cell lines was induced with UV-irradiation and monitored by Annexin-V-FITC Apoptosis detection Kit from PharMingen (Becton Dickinson Company, CA USA) and cultured with DC at a DC-tumor ratio of 1∶1 for 18 hours. Tumor-loaded DC were then used to stimulate autologous T CD4 naïve cells at a S:R ratio of 1∶4. After 4 days of culture, the presence of IL-17-producing T cells was monitored by ELISA after stimulation with anti-CD3 coated beads (T cell activation kit, MiltenyiBiotec) for additional 24 h.

### Statistical analysis

All results are expressed as mean ± SD, whereas the statistical analysis was performed by Mann-Whitney test.
